# Pulling away from the trigger: the influences of purpose in life and self-affirmation on decisions to shoot

**DOI:** 10.3389/fcogn.2024.1397643

**Published:** 2024-09-17

**Authors:** Kayla A. Burd, Anthony L. Burrow, Max Guyll

**Affiliations:** ^1^Department of Psychology, University of Wyoming, Laramie, WY, United States; ^2^Department of Psychology, Cornell University, Ithaca, NY, United States; ^3^School of Interdisciplinary Forensics, Arizona State University, Glendale, AZ, United States

**Keywords:** shooting decisions, bias, purpose in life, self-affirmation, race

## Abstract

**Introduction:**

Recent data suggests significant racial disparities in police killings in the United States: Much research finds that Black men are killed by police officers at higher rates than White men, and many individuals killed by police have been unarmed.

**Method:**

Toward addressing psychological mechanisms at play in these complicated decision contexts, the current study tested the effectiveness of two writing tasks at reducing the unjustified shooting of unarmed targets using a virtual shooting-decision platform. Participants wrote either about their sense of purpose, self-affirming values, or a control topic and then played a first-person shooter video game, which randomly presented pictures of Black and White armed and unarmed targets. Participants were instructed not to shoot unarmed targets and to shoot armed targets.

**Results:**

Results indicated that relative to controls, writing about either purpose or self-affirming values reduced the probability of shooting unarmed targets, without negatively impacting shooting decision reaction time.

## Introduction

Police officers, like other citizens, face threats, and stressors of everyday life. However, police officers face additional threats particular to their line of work. Evidence suggests that the leading stressors for police officers are the fear of killing someone while on duty and the fear of being attacked themselves (Arial et al., 2010; Ma et al., 2015; Violanti and Aron, 1995). These stressors and perceptions of threat relate to officer-involved shootings. For instance, if an officer believes that they or a colleague might be harmed, the officer may decide to shoot a suspect. In ambiguous contexts, when spilt-second decisions are more difficult, decision errors are more likely, and unarmed suspects may be harmed. Subjective perceptions of threat influence officer decisions to shoot, which can be influenced by implicit and explicit racial biases.

Recent field data suggests significant racial disparities in police shootings. In 2016, Black males between 15–24 years of age were nine times more likely to be killed by police officers compared to other Americans (Swaine and McCarthy, 2017). While insight into the situational factors that underlie shooting decisions is lacking (Burch and Cave, 2017), a growing number of laboratory and field studies suggest tacit beliefs may contour these decisions in systematic and racially biased ways (Correll et al., 2014). Studies have shown that police officers, like the general public, hold implicit and/or explicit racial biases, which can impact decisions to shoot (Correll et al., 2014). Thus, it is important to examine basic psychological processes to understand the range of factors that might influence these behaviors. In other contexts, purpose in life and self-affirmation have been shown to improve comfort with diversity and reduce reactivity to threat (for a review, see Burd and Burrow, 2017). In the current study, we examine these constructs in the context of a first-person shooter task to test whether purpose in life or self-affirmation may impact basic psychological processes that influence shooting decisions for Black and White targets.

### Racial biases in shooting decisions

Social psychological research has begun to examine the impact of implicit racial biases on shooting decisions involving minority suspects (e.g., Correll et al., 2002, 2006; Greenwald et al., 2003). Studies examining shooting decisions find that individuals exhibit racial bias in accuracy and reaction time (e.g., Correll et al., 2002, 2006; Greenwald et al., 2003). Participants in these studies mistakenly shoot unarmed Black men holding harmless objects (e.g., cell phones, wallets) more than unarmed White men holding such objects (e.g., Correll et al., 2002, 2006; Greenwald et al., 2003). Further, individuals fire at armed Black men more quickly than armed White men and are slower to fire at unarmed White men compared to unarmed Black men (e.g., Correll et al., 2002, 2006; Greenwald et al., 2003). This tendency has been described as *shooter bias*. Taken together, these findings suggest that individuals have a lower decision criterion for shooting Black vs. White men (Correll et al., 2002), and have trouble distinguishing weapons from harmless objects when targets are Black (Greenwald et al., 2003). Related research has explored individuals' associations between ethnicities and various objects. Some have found that Black faces were more closely associated with handguns and sports objects than White faces (Judd et al., 2004), whereas Payne (2001, 2005) found that participants are more likely to mistake tools as guns following the presentation of Black compared to White faces, and are more likely to pair “bad” with Black and “good” with White faces (Payne, 2005). There is growing recognition that racial biases and negative stereotypes predict inaccuracies in shooting decisions for Black and White targets (e.g., Correll et al., 2002, 2006).

***Causes and correlates of shooter bias***

The strength of one's biases and negative stereotypes of minorities impacts shooting decisions (e.g., Sadler et al., 2012) and corresponding reaction times (e.g., Correll et al., 2007). Research suggests that explicit prejudice and personal endorsement of negative stereotypes regarding Black individuals tend to be unrelated to shooter bias (Correll et al., 2002; Payne, 2001). However, measures of negative cultural stereotypes regarding Black individuals are related to shooter bias (Correll et al., 2002). Evidence suggests that prejudice is related to automatic processes like shooter bias for individuals who have low motivation to control prejudices (Payne, 2001), and personal prejudice has been shown to be positively related to negative cultural stereotypes in individuals who are not motivated to control prejudice (Correll et al., 2002). Theorists have suggested that participants may be using negative stereotypes regarding Black individuals to disambiguate whether suspects are armed or unarmed (Correll et al., 2002; Payne, 2001).

Broadly speaking, researchers argue that response inhibition is the key to such decisions: Many individuals experience implicit bias, but those who feel threatened and are *unable* to inhibit the improper response are more likely to exhibit shooter bias (Correll et al., 2006). Importantly, research suggests these biases are exacerbated among participants exhibiting low cognitive control. Although individuals with high or low cognitive control can be equally biased, those with high control are less likely to express their biases in behaviors and judgments (Payne, 2005). The effects of racial bias on shooting decisions may be further exacerbated by depletion and fatigue (Ma et al., 2013), and the need to make split-second decisions (Payne, 2001, 2006).

***Perceptions of threat stemming from racial biases***

Many factors impact police officers' decisions to use force, including encounter and suspect characteristics (Bolger, 2015). Encounter characteristics involve the interaction between the officer and a suspect and are related to an increased likelihood of force being used. The likelihood that force is used may also depend on the characteristics of a suspect. For example, force is more likely when suspects are male, minorities, from a lower social class, or hostile (Bolger, 2015). Importantly, both encounter and suspect characteristics may impact police officers' *subjective* perceptions of the interaction and can relate to officers' perceptions of threat.

Threat, and subjective perceptions of threat, can take many forms and can impact behavior in important ways. One source of threat that may be pertinent to police officers, among others, is discomfort with diversity. While longstanding psychological theory predicts that interracial contact reduces racial bias (e.g., Allport, 1954), recent empirical research paints a more nuanced picture. Individuals perceive greater personal threat in more ethnically diverse contexts (e.g., Burrow and Hill, 2013; Outten et al., 2012). For example, research suggests that perceptions of increasing diversity induce implicit and explicit racial biases in White Americans (Craig and Richeson, 2014). Thus, actual or perceived changes in demographic diversity may cause individuals to become *more* rather than *less* racially biased. These perceptions of threat can impact all citizens, but they may play an important role in understanding improper shootings by police officers.

Implicit and explicit racial bias, prejudice, and stereotyping have been assessed in a number of ways. For example, implicit racial prejudice has been measured using the race IAT (Greenwald et al., 1998) and stereotype-specific IATs (e.g., Amodio and Devine, 2006), among others. However, given prior research demonstrating that purpose in life writing tasks may help to inhibit impulsivity (Burrow and Spreng, 2016) as well as increase comfort with diversity (Burrow et al., 2014a), whereas research examining self-affirmation finds that such interventions might facilitate self-regulation (Loseman and van den Bos, 2012) and promote self-control (Schmeichel and Vohs, 2009), in the current research, we chose measures that might target both implicit and explicit motivations, namely, the Internal and External Motivation to Respond without Prejudice Scales (Plant and Devine, 1998). Additionally, to capture racial prejudice in a way that might be less susceptible to participant demand characteristics, we also included the Color-Blind Racial Attitudes Scale (CoBRAS; Neville et al., 2000).

### Expertise and training: improving shooting decisions

Training and experience with firearms might attenuate the impact of racial biases in shooting decisions. Research comparing shooting decisions among officers and community members indicates that, although both samples are racially biased, police officers make more accurate shooting decisions than civilians (Correll et al., 2007). In this work, officers were faster than civilians in their decisions to shoot and were better able to differentiate among armed and unarmed suspects compared to community members. Still, community members and police officers made faster decisions to shoot armed Black suspects compared to armed White suspects, and were slower when making decisions about unarmed Black suspects compared to unarmed White suspects. Thus, in this laboratory-based study, officers did not exhibit racial biases in shooting decision accuracy, but evidenced biases in response latencies. In the line of duty, these delays in determining that an unarmed suspect is in fact unarmed pose a real threat to unarmed suspects. In every second that passes between initial contact with a suspect and the decision that a suspect is unarmed, the decision context may become clouded by additional external factors (e.g., bystanders may appear, other suspect behaviors may be interpreted as threatening). These delays likely exacerbate the impact of racial biases on officer perceptions of threat, in turn putting innocent suspects at increased risk.

Building on research which demonstrates that exposure to counter-stereotypic examples can reduce automatic stereotyping, Plant and colleagues exposed undergraduates (Plant et al., 2005) and police officers (Plant and Peruche, 2005) to a training task aimed at reducing such automatic responses. Participants were presented with Black and White faces, over which guns or harmless objects were superimposed. Participants were asked to actively press a button to shoot for trials featuring a weapon, and to actively make the decision not to shoot for trials involving harmless objects. Over many trials, participants improved in their decisions to shoot and exhibited fewer errors. Further, the largest improvement in performance was found for trials involving Black faces paired with neutral objects. These findings suggest that repeated exposure to Black and White faces, wherein race was unrelated to whether a harmless object or weapon was presented, can reduce biases in shooting decisions through a reduction in automatic processes. Thus, there is some evidence that biases may be reduced through training. Several questions remain; what basic psychological processes are at play during these split-second shooting decisions, and what psychological constructs can be harnessed to reduce perceptions of threat, and in turn, improve shooting decisions?

## Purpose in life and self-affirmation: protection from perceived threat

There is some evidence that experience and training may attenuate the impact of racial biases on decisions to shoot. However, the findings are mixed, and less is known regarding by what mechanism experience and training might improve decision accuracy in this context. It is important to explore psychological constructs that might be harnessed to reveal by what mechanism shooting decisions may be improved. Recent theory and research suggest that *purpose in life* and *self-affirmation* may be relevant in this context. Both purpose in life and self-affirmation offer protective benefits, helping individuals to feel more comfortable with diversity, reducing individuals' reactivity to and recovery from stress, and improving accuracy in judgments (for a review, see Burd and Burrow, 2017). Thus, purpose in life and self-affirmation may provide valuable insight into the psychological mechanisms at play during these complex decisions.

### Purpose in life

Purpose in life is a “central, self-organizing life aim” which provides meaning (McKnight and Kashdan, 2009, p. 242). Purpose allows individuals to situate themselves in broader social contexts (Bronk, 2011) and to imagine their ideal future selves. Research indicates that purpose in life helps reduce reactivity to and recovery from stress and threat (e.g., Ishida and Okada, 2006; Schaefer et al., 2013), inhibits impulsivity (Burrow and Spreng, 2016), and is related to increased comfort with diversity (Burrow et al., 2014a). Purpose helps buffer against negative affect during times of uncertainty, discomfort, or change (e.g., Burrow et al., 2014a), and is generally related to increased wellbeing (e.g., Burrow et al., 2014b). Further, much research suggests that purpose in life is negatively associated with fear of one's own death and death avoidance (Ardelt, 2003, 2007; Drolet, 1990). Purposeful individuals primed to think about their own deaths do not experience increased death anxiety (Routledge and Juhl, 2010).

While dispositional purpose in life has many positive correlates, it is important to note that such outcomes can be achieved through purpose interventions as well. For instance, in one study researchers asked individuals to write about their sense of purpose in life and found that these individuals, compared to those who wrote about a control topic, were more comfortable when confronting diversity (Burrow et al., 2014a). Further, writing about one's sense of purpose has also been shown to decrease antisocial behaviors in impoverished adolescents (Machell et al., 2016) and to increase confidence in mock legal investigators (Burd et al., 2016).

Thus, purpose in life is implicated in many positive outcomes that may relate to death anxiety and decisions to shoot. For example, when confronted by others, individuals may perceive increased threat, interpreting harmless objects as weapons. Importantly, automatic processing maybe exacerbated when individuals confront diverse others. Purpose in life may reduce perceptions of threat, death anxiety, and impulsivity, giving individuals time to interpret harmless objects as such.

### Self-affirmation

Evidence shows that affirming the self in a context unrelated to the threat reminds people of who they are (Sherman and Cohen, 2006) by drawing on alternative resources of self-worth (Cohen et al., 2000; Sherman and Cohen, 2002). In the face of threat, individuals seek to maintain a positive self-concept and are motivated to protect their sense of self-worth and integrity (e.g., Sherman and Cohen, 2006; Steele, 1988). Individuals may respond directly or indirectly, and sometimes in a defensive, unproductive manner (Sherman and Cohen, 2006). However, self-affirmation can help individuals cope with such threats. Self-affirmation increases individuals' perceptions of their self-resources, which in turn is associated with lower stress appraisal (Creswell et al., 2005). In addition, self-affirmation can facilitate self-regulation (Loseman and van den Bos, 2012), promote self-control when one is cognitively depleted (Schmeichel and Vohs, 2009), and may reduce perceptions of stress (Sherman et al., 2009). Further, self-affirmation interventions have been shown to reduce individuals' implicit racial biases (Frantz et al., 2004), and mortality salience (Schmeichel and Martens, 2005). Police officers may experience mortality salience in their daily lives, and in addition, may experience stereotype threat when encountering diverse suspects (Richardson and Goff, 2014).

### Purpose in life, self-affirmation, and other interventions

Given that purpose in life and self-affirmation can help reduce individuals' reactions to, and promote recovery from, stressful (e.g., Fogelman and Canli, 2015; Schaefer et al., 2013) or threatening events, and can even prevent perceptions of stress (Sherman et al., 2009), both might serve as helpful interventions to law enforcement officers by mitigating racial biases, which may relate to shooting decisions. Purpose helps buffer against negative affect during times of uncertainty, discomfort, or change (e.g., Burrow et al., 2014a), and is generally related to increased wellbeing (e.g., Burrow et al., 2014b). Self-affirmation increases individuals' perceptions of their self resources, which in turn, is associated with lower stress appraisal (Creswell et al., 2005). Further, self-affirmation can facilitate self-regulation (Loseman and van den Bos, 2012) and promote self-control when one is cognitively depleted (Schmeichel and Vohs, 2009). Taken together, purpose in life and self-affirmation may reduce officers' perceptions of threat, which in turn, may increase shooting decision accuracy, particularly for minoritized individuals.

To date, little research has empirically examined the conceptual overlap and/or differences between purpose in life and self-affirmation. One study investigated the relation between a self-affirmation intervention and eudemonic wellbeing, which included questions pertaining to purpose in life (Nelson et al., 2014). The self-affirmation intervention was shown to influence wellbeing, suggesting that self-affirmation interventions might help to cultivate purpose in life. However, in this context, wellbeing was measured using four items, only one of which targeted purpose in life, and the research did not pit purpose in life and self-affirmation interventions against each other (Nelson et al., 2014). Though some scholars have sought to theoretically examine the similarities and differences between purpose in life and self-affirmation (Burd and Burrow, 2017), no known research has done so experimentally, which was a central aim of the present research.

Many have theorized about, and empirically examined, interventions aimed at reducing prejudice and stereotyping against outgroup members (for a review, see FitzGerald et al., 2019; Lai et al., 2014). Innovative interventions have included, for example, training participants to approach outgroup members using a joystick (Kawakami et al., 2007), establishing a connection between the self and outgroup members (e.g., Woodcock and Monteith, 2013), counterstereotype conditioning, and the promotion of multiculturalism, among others (for a review, see FitzGerald et al., 2019; see also Lai et al., 2014). Generally, interventions that include counterstereotype conditioning have demonstrated success at reducing implicit prejudice and stereotyping (e.g., Woodcock and Monteith, 2013; see also FitzGerald et al., 2019), whereas those that make connections between the self and outgroup members have proven less successful at reducing stereotyping (e.g., Woodcock and Monteith, 2013).

The current study, in its investigation into the possibility that purpose in life and self-affirmation interventions might reduce racial bias in the context of a first-person shooter video game, differs from these prior interventions in at least one important way. In general, the interventions described above tend to tackle racial bias rather directly (e.g., directly exposing participants to photographs of Black and White individuals; Kawakami et al., 2007). However, the purpose in life and self-affirmation tasks utilized in the current research make no direct connection to race, as participants responded to either writing task in an open-ended manner, with no direct link to race. Therefore, the current writing tasks might be conceptualized as less direct, with the potential for more nuance given that participants could respond in any way they chose. Thus, the current research builds on and extends prior research examining bias mitigation and tests these writing interventions (i.e., purpose in life and self-affirmation) in a novel context using a first-person shooter video game behavioral measure.

## Study overview

Taken together, an experimental test of the effects of purpose in life and/or self-affirmation may provide valuable insight into the psychological mechanisms that may impact shooting decisions and response latencies. Perceptions of threat relate to police decisions to shoot, and extant literature suggests that officers perceive increased threat for Black compared to White suspects. The aim of the current study was to test the potential impact of purpose in life and self-affirmation writing tasks on the basic psychological processes that may be involved in police shooting decisions. If perceptions of threat drive these decisions to shoot, then we should expect shooting decision accuracy to be improved for those who affirm the self or write about their purpose in life before participating in a first-person shooter task. Further, if perceptions of threat are greater when a target is armed, and are exacerbated by racial biases against Black targets, we would expect these interventions to have a greater influence for Black compared to White targets.

Self-affirmation and purpose in life operate to reduce threat across many contexts, and in turn, may reduce defensive behaviors. For instance, research suggests that self-affirmation lowers threat appraisal (Creswell et al., 2005), promotes self-regulation (Loseman and van den Bos, 2012) and self-control (Schmeichel and Vohs, 2009), and reduces implicit racial biases (Frantz et al., 2004), while purpose in life is associated with increased comfort with diversity and reduced negative affect (Burrow et al., 2014a), which in turn may also promote decision accuracy. Thus, it was hypothesized that those who affirm the self or write about one's sense of purpose in life will make more accurate shooting decisions (will be less likely to improperly shoot unarmed suspects) in a first-person shooter video game task relative to individuals who write about a control topic, and that such effects will be greater for Black compared to White targets. Lastly, it is hypothesized that participants in the purpose in life writing condition will respond more slowly than those in the self-affirmation and control conditions based on research demonstrating the purpose in life is associated with lower impulsivity, which may increase the time participants take to make shooting decisions (Burrow and Spreng, 2016).

Given prior research showing that purpose in life promotes comfort with diversity (e.g., Burrow et al., 2014a), we expected an interaction between writing task and race. The current research also examines whether purpose in life and/or self-affirmation writing interventions might relate to several potential underlying mechanisms (e.g., internal and external motivations to respond without prejudice) that may explain the potential for a relationship between these writing interventions and shooting decision accuracy. However, given that prior research has not to date examined whether such writing tasks may influence these underlying mechanisms, no hypotheses were made regarding these constructs.

## Methods

### Participants

An a priori power analysis suggested a required sample size of 492 participants to achieve a power of 0.80 for detecting a small effect size of *f* = 0.10, when employing a significance criterion of *p* = 0.05 alpha (G^*^Power 3; see Faul et al., 2007). Five hundred thirty-eight adults (*Mage* = 19.61, *SD* = 1.77, *Range*: 18–35, age unknown for 1 participant) participated in the experiment in exchange for course credit or on a volunteer basis without compensation. Subjects were recruited from two large universities in the US, one in the Northeast (*n* = 96), the other in the Midwest (*n* = 442). Seventeen participants were excluded from analyses due to a file corruption, knowledge of the true nature of the study, or experimenter error (final *N* = 521). The sample was 61.5% male (38.3% female, 0.2% “other,” data missing for 2 individuals), and primarily White (75.8% White, 9.6% Asian/Pacific Islander, 7.7% Hispanic, 3.7% Black, 3.3% other).

### Design

The experiment employed a 3 (Writing task: Purpose in Life vs. Self-Affirmation vs. Control) × 2 (Race: Black vs. White) × 2 (Weapon: Armed vs. Unarmed) mixed design with repeated measures on the factors of *race* and *weapon*.

### Procedure

All participants completed the experiment independently. First, participants were greeted by an experimenter and taken to a private laboratory space and asked to sit at a desktop computer. After receiving basic instructions and providing consent, participants completed several individual difference measures. Next, participants received further brief instructions and then completed one of the randomly assigned writing tasks (control vs. purpose in life vs. self-affirmation). Immediately after finishing the writing task, participants engaged in a first-person shooter video game. After, participants completed the individual difference measures for a second time, and lastly, answered several demographic questions. Participants were then debriefed and thanked for their participation.^1^

The individual difference measures administered (e.g., Internal and External Motivations to Respond without Prejudice Scales, IMS/EMS; Plant and Devine, 1998) are typically conceptualized as relatively stable traits. However, in the current study we were interested in exploring whether purpose in life and/or self-affirmation writing tasks could effect change on these traits. Thus, these individual difference measures were administered both before and after participants completed the writing task and engaged with the first-person video game, and analyses detailed below examined pre- and post-writing task scores.

### Manipulations

Participants were randomly assigned to one of three experimental conditions (purpose in life vs. self-affirmation vs. control). Participants in the purpose in life writing condition (*n* = 173; Burrow and Hill, 2013) were asked to write for 10 min in response to the following prompt:

*Please take 10 min to think about your sense of purpose in life. Really reflect on the idea of purpose. When you are ready, please describe your sense of purpose (e.g., What is your purpose and where did it come from?)*.

*If you do not have a sense of purpose, or are unsure about what it might be, please take a few minutes to consider the idea of purpose in life and what it would mean for you to have a purpose. Really reflect on what it would mean in your life. When you are ready, describe as best as you can what you think it would mean to you*.

Participants in the self-affirmation writing condition (*n* = 173) first ranked six values in order of personal importance (business, art/music/theater, social life/relationships, science/pursuit of knowledge, religion/morality, and government/politics; e.g., Steele and Liu, 1983), from 1 (*most personally* important) to 6 (*least personally* important). Next, participants wrote for 10 min in response to the following prompt:

“*Please write for 10 min about your* ***most*** *important value from above. Why is this value most important to you? Why is this value so meaningful?”*

Participants in the control condition (*n* = 175) completed the same values ranking task as in the self-affirmation condition, but instead wrote about their least important value in response to the following prompt:

“*Please write for 10 min about your* ***least*** *important value from above. Why might this value be important to others? Why is this value so meaningful to others?”*

These methods are commonly used in research examining the effects of self-affirmation (e.g., Creswell et al., 2013; Martens et al., 2006).

### Materials

**Video game**. The current study employed a first-person shooter video game administered via E-Prime on desktop computers utilizing identical procedures to Correll et al. (2002). Participants engaged in 16 practice trials and 100 test trials of the video game; 25 trials for each of the 2 (Race: Black vs. White) × 2 (Weapon: Armed vs. Unarmed) within-subjects portion of the design. Thus, participants viewed 100 separate images portraying men (White or Black) who were armed (holding a gun) or unarmed (holding some harmless object, like a cellphone or wallet). Each target image of a suspect was randomly presented and displayed against one of several different backgrounds (e.g., a mall, a street, a park). For each trial, participants viewed a random number (0–3) of backgrounds with no suspect present, and the images remained on the screen for a random period of time (ranging from 500 ms−800 ms). Next, a final background would appear, for a random amount of time. Then, this background would be replaced with an image of a suspect against this same background.

Participants were instructed to “shoot” at armed individuals by pressing the “p” key, and told to actively “not shoot” by pressing the “q” key as quickly as possible. Participants had 630 ms to make a shooting decision before the trial timed out. To incentivize both speed and accuracy, participants earned 10 points for correctly shooting armed targets and 5 points for correctly choosing not to shoot unarmed targets. Further, participants lost 20 points for shooting unarmed targets, and 40 points for failing to shoot armed targets. In addition, participants lost 10 points if they failed to respond within 630 ms (see Correll et al., 2002). The point structure here was intended to mirror trade-offs that police officers make in the field (i.e., police seek to reduce shooting errors of unarmed suspects, but are motivated to avoid being harmed themselves, see Correll et al., 2002), and to reduce trials with non-responses. Participants received feedback (visual and auditory), and saw their cumulative points displayed on the screen as they progressed through each trial.

### Measures

**Shooting decisions and reaction time**. Participants' shooting decisions were recorded for each of the practice and test trials along with shooting decision reaction times. Trials wherein participants timed out (i.e., did not make a shooting decision within 630 ms) were excluded from this analysis. Further, only participants' test trials were analyzed.

**Motivations to respond without prejudice**. The Internal and External Motivations to Respond without Prejudice Scales (IMS/EMS; Plant and Devine, 1998) were used to assess individuals' motivations to respond without prejudice based on self-imposed standards (internal) and based on standards imposed by others (external). Each scale contains five items, and the ten total items were intermixed. Responses range from 1 (*strongly disagree*) to 7 (*strongly agree*). Sample items include “*I try to hide any negative thoughts about Black people in order to avoid negative reactions from others”* (external motivation; Cronbach's α = 0.77 pre-writing and 0.86 post-writing) and “*Being non-prejudiced toward Black people is important to my self-concept”* (internal motivation; Cronbach's α = 0.76 pre-writing and 0.80 post-writing). Higher numbers indicate more motivation to respond without prejudice for each respective motivation.

**Colorblind attitudes**. The Color-Blind Racial Attitudes Scale (CoBRAS; Neville et al., 2000) is a 20-item measure that assesses colorblind attitudes. Sample items include “*Racial problems in the U.S. are rare, isolated situations*” and “*White people in the U.S. have certain advantages because of the color of their skin*” (reverse-coded). Participants rated each item from 1 (*strongly disagree*) to 7 (*strongly agree*). Individuals with higher scores deny that racism has structural components, and that racism creates advantages for White individuals and disadvantages minorities (Cronbach's α = 0.91 pre-writing and 0.92 post-writing).

**Purpose in life**. Purpose was measured using nine items from the Ryff Scales of Psychological Well-Being (Ryff, 1989). Sample items include “*I enjoy making plans for the future and working to make them a reality*,” and “*My daily activities often seem trivial and unimportant to me,”* (reverse-coded) on a scale from 1 (*strongly disagree*) to 7 (*strongly agree*), with higher numbers indicating more purpose in life (Cronbach's α = 0.81 pre-writing and 0.84 post-writing).

**Moral foundations**. The Moral Foundations Questionnaire (MFQ-30; Graham et al., 2008) was utilized to measure individuals' reliance on five moral foundations (care/harm, fairness/cheating, loyalty/betrayal, authority/subversion, sanctity/degradation). Responses range from 1 (*not at all relevant/strongly disagree*) to 6 (*extremely relevant/strongly agree*). Respondents are asked to consider what attributes are important to them when deciding whether something is right or wrong. Sample items include, “*whether or not someone suffered emotionally*” (care/harm; Cronbach's α = 0.57 pre-writing and 0.65 post-writing), “*whether or not someone showed a lack of respect for authority*” (authority/subversion; Cronbach's α = 0.69 pre-writing and 0.71 post-writing), “*whether or not some people were treated differently than others*” (fairness/cheating; Cronbach's α = 0.58 pre-writing and 0.67 post-writing), “*whether or not someone's action showed love for his or her country*” (loyalty/betrayal; Cronbach's α = 0.66 pre-writing and 0.74 post-writing), and “*whether or not someone violated standards of purity and decency*” (sanctity/degradation; Cronbach's α = 0.70 pre-writing and 0.78 post-writing). Higher scores on each of the five subscales indicates stronger endorsement of these principles when considering whether someone thing right or wrong.

**Affect**. Participants completed the Positive and Negative Affect Schedule (PANAS; Watson et al., 1988). Participants were asked to indicate how they felt, *right now*, using a scale ranging from 1 (Very slightly to not at all) to 5 (Extremely) for a large variety of emotions (e.g., anger, anxiety, calm, disgust, surprised, upset). Ten items each were recorded for both positive (Cronbach's α = 0.87) and negative affect (Cronbach's α = 0.86), and positive and negative affective terms were intermixed.

**Demographic questionnaire**. Participants responded to several demographic questions regarding age, sex, ethnicity, education, and political orientation on a Likert-type scale ranging from 1 (E*xtremely Liberal*) to 7 (*Extremely Conservative*).

## Results

### Shooting decisions

#### Purpose in life vs. self-affirmation

**Figure 1:**
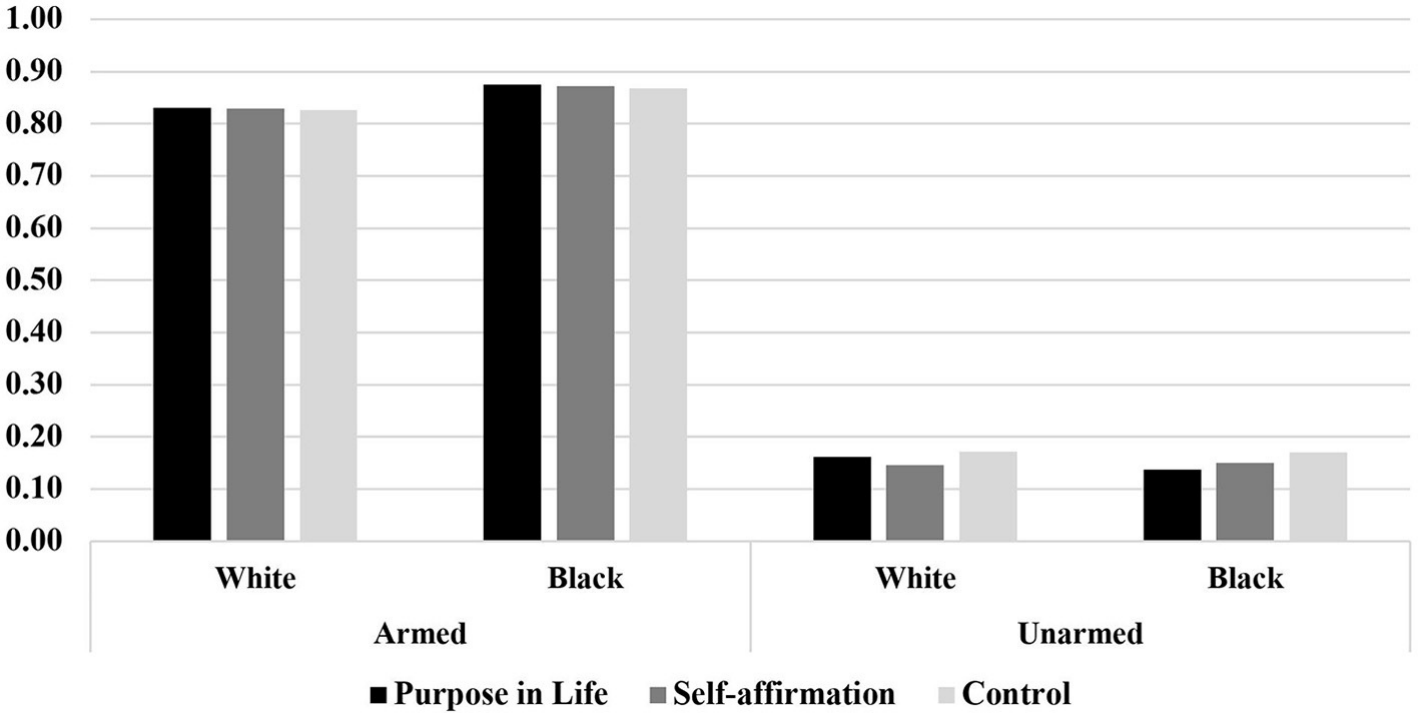
Mean probability of shooting targets by writing task, race, and weapon.

To test whether the writing tasks impacted participants' shooting decisions differentially for White and Black targets who were armed or unarmed, a 3 (Writing task: Purpose in Life vs. Self-Affirmation vs. Control) × 2 (Race: Black vs. White) × 2 (Weapon: Armed vs. Unarmed) mixed model binary logistic regression was conducted, with repeated measures on the factors of *race* and *weapon*. Participants' responses were coded “1” if they made a decision to shoot and “0” if they made a decision to *not* shoot (see Table 1, Figure 1). No evidence was found of a three-way interaction, *F*_(2, 44, 712)_ = 2.09, *p* = 0.12.

**Table 1:** Effect of writing task, target race, and weapon presence on probability of shooting.

					**Effect size**		
		***df***	***F***	***p***	***η^2^***	**90% CI**	
Model 1	Writing task (PIL vs. SA vs. Control)	2	2.57	0.08	0.00	0.00	0.00
	Weapon	1	16,858.18	< 0.001	0.00	0.00	0.00
	Race	1	28.62	< 0.001	0.27	0.27	0.28
	Writing task × Weapon	2	7.05	0.001	0.00	0.00	0.00
	Writing task × Race	2	1.45	0.24	0.00	0.00	0.00
	Weapon × Race	1	56.72	< 0.001	0.00	0.00	0.00
	Writing task × Weapon × Race	2	2.09	0.12	0.00	0.00	0.00
	Error *df*	44,712					
Model 2	Writing task (Writing Tasks vs. Control)	1	4.83	0.03	0.00	0.00	0.00
	Weapon	1	15,109.65	< 0.001	0.25	0.25	0.26
	Race	1	27.37	< 0.001	0.00	0.00	0.00
	Writing task × Weapon	1	13.96	< 0.001	0.00	0.00	0.00
	Writing task × Race	1	0.14	0.71	0.00	0.00	0.00
	Weapon × Race	1	47.77	< 0.001	0.00	0.00	0.00
	Writing task × Weapon × Race	1	0.83	0.36	0.00	0.00	0.00
	Error *df*	44,716					

Analyses revealed significant main effects of race and weapon, while no significant main effect was found for writing task (see Figure 1). Participants were significantly more likely to shoot Black (*M* = 0.52, *SE* = 0.01) compared to White targets, *M* = 0.49, *SE* = 0.00, *t*_(44, 712)_ = 5.35, *p* < 0.001, *d* = 0.05, 95% CI [0.03, 0.07] as well as armed (*M* = 0.85, *SE* = 0.00) vs. unarmed (*M* = 0.16, *SE* = 0.00) targets (*t*_(44, 712)_ = 129.84, *p* < 0.001, *d* = 1.23, 95% CI [1.21, 1.25]) and (see Table 2).

**Table 2:** Means (and standard errors) for probability of shooting and shooting decision reaction times as a function of writing task, weapon, and target race.

**Condition**			**Probability of shooting**	**Reaction times (ms)**
Control	Armed	White	0.83 (0.01)	492.18 (1.10)
		Black	0.87 (0.01)	490.50 (1.09)
	Unarmed	White	0.17 (0.01)	522.52 (1.14)
		Black	0.17 (0.01)	526.83 (1.17)
Purpose in life	Armed	White	0.83 (0.01)	491.99 (1.11)
		Black	0.87 (0.01)	489.49 (1.10)
	Unarmed	White	0.16 (0.01)	523.02 (1.15)
		Black	0.14 (0.01)	528.46 (1.18)
Self-affirmation	Armed	White	0.83 (0.01)	494.93 (1.11)
		Black	0.87 (0.01)	492.00 (1.10)
	Unarmed	White	0.15 (0.01)	524.03 (1.15)
		Black	0.15 (0.01)	528.07 (1.19)

A significant interaction was found between race and weapon (see Table 1). Analyses revealed no differential effect for the shooting Black or White unarmed targets, *t*_(44, 712)_ = 1.52, *p* = 0.13, *d* = 0.02, 95% CI [−0.01, 0.05]. However, participants were significantly more likely to shoot Black targets (*M* = 0.87, *SE* = 0.00) compared to White targets when armed (*M* = 0.83, *SE* = 0.00, *t*_(44, 712)_ = 9.27, *p* < 0.001, *d* = 0.12, 95% CI [0.10, 0.15] (see Figure 1).

Further, a significant interaction was found between writing task and weapon (see Table 1). Analyses revealed no effect of the writing tasks as compared to the control condition for armed targets, *p*s > 0.25. However, purpose in life (*M* = 0.15, *SE* = 0.00) reduced the shooting of unarmed targets compared to the control condition, *M* = 0.17, *SE* = 0.00, *t*_(44, 712)_ = −3.43, *p* = 0.001, *d* = 0.06, 95% CI [0.03, 0.09]. In addition, self-affirmation (*M* = 0.15, *SE* = 0.00) reduced the shooting of unarmed targets compared to the control condition, *M* = 0.17, *SE* = 0.00, *t*_(44, 712)_ = −3.73, *p* < 0.001, *d* = 0.06, 95% CI [0.03, 0.10] (see Figure 1). However, no significant difference was found between purpose in life and self-affirmation for armed (*p* = 0.70) or unarmed targets, *p* = 0.76.

#### Writing tasks vs. control condition

We next collapsed across writing interventions to determine whether the writing interventions generally might influence participants' shooting decisions. A 2 (Writing task: Intervention vs. Control) × 2 (Race: Black vs. White) × 2 (Weapon: Armed vs. Unarmed) mixed model binary logistic regression was conducted, with repeated measures on the factors of *race* and *weapon*. As discussed above, participants' responses were coded “1” if they made a decision to shoot and “0” if they made a decision to *not* shoot. As above, no evidence was found of a three-way interaction, *F*_(1, 44, 716)_ = 0.83, *p* = 0.36, see Table 1.

Analyses revealed significant main effects of writing task, weapon, and race. Participants who completed either writing task were significantly less likely to shoot targets (*M* = 0.50, *SE* = 0.004) compared to those in the control condition, *M* = 0.52, *SE* = 0.006, *t*_(44, 716)_ = 2.20, *p* = 0.03, *d* = −0.02, 95% CI [0.00, 0.04]. In line with the results above, participants were significantly more likely to shoot Black (*M* = 0.53, *SE* = 0.01) compared to White targets, *M* = 0.49, *SE* = 0.01, *t*_(44, 716)_ = 5.24, *p* < 0.001, *d* = 0.05, 95% CI [0.03, 0.07], as well as armed (*M* = 0.85, *SE* = 0.00) vs. unarmed (*M* = 0.16, *SE* = 0.00) targets [*t*_(44, 716)_ = 191.10, *p* < 0.001, *d* = 1.81, 95% CI (1.79, 1.83)].

A significant interaction was found between race and weapon. Mirroring findings above, analyses revealed no differential effect for the shooting Black or White unarmed targets, *t*_(44, 716)_ = 1.18, *p* = 0.24, *d* = 0.02, 95% CI [−0.01, 0.04]. However, as above, participants were significantly more likely to shoot Black targets (*M* = 0.87, *SE* = 0.00) compared to White targets when armed [*M* = 0.83, *SE* = 0.00, *t*_(44, 716)_ = 8.70, *p* < 0.001, *d* = 0.11, 95% CI (0.09, 0.14)]. No evidence was found of an interaction between race and writing task, *p* = 0.71.

Further, a significant interaction was found between writing task and weapon. As above, analyses revealed no effect of the writing tasks as compared to the control condition for armed targets, *p*s > 0.27. However, the writing tasks (*M* = 0.15, *SE* = 0.00) reduced the shooting of unarmed targets compared to the control condition, *M* = 0.17, *SE* = 0.00, *t*_(44, 716)_ = −4.09, *p* < 0.001, *d* = 0.05, 95% CI [0.02, 0.08].

**Signal-detection analyses**. Previous research has found that participants are equally able to discriminate amongst Black and White targets but have a lower decision threshold for Black vs. White targets (e.g., Correll et al., 2002, Study 2; Correll et al., 2007, Study 1). Signal-detection analyses allow for estimates of participants' ability to discriminate amongst targets who are armed and unarmed (*d'*), and their shooting decision threshold (*c*).

We calculated participants' ability to discriminate between armed and unarmed targets (*d'*) and their shooting decision threshold (*c*) for Black and White targets. A Hautus (1995) correction was applied to address proportions equal to 0 and 1. Proportions of 0 and 1 would yield infinite values. A 3 (Writing task: Purpose in Life vs. Self-Affirmation vs. Control) × 2 (Race: Black vs. White) mixed model was conducted, with repeated measures on *race* to test whether *d'* or *c* varied for Black or White targets based on participant Writing Task. Higher *d'* values refer to how much more likely a participant would be to shoot and armed compared to unarmed target, while *c* refers to the threshold (or criterion) a participant used in deciding whether to shoot. Greater *c* values would imply a participant was less likely to shoot overall. Analyses revealed that Writing Task did not predict *d'* or *c* for Black or White targets, nor was there any evidence of an interaction between Writing task and either *d'* or c, *ps* ≥ 0.15 (see Table 3).

**Table 3:** Effect of writing task on sensitivity and threshold.

					**Effect size**		
		***df***	***F***	***p***	***η^2^***	**90% CI**	
Model 1	Discriminability (*d'*)						
	Race	1	70.17	< 0.001	0.12	0.08	0.16
	Writing task	2	0.96	0.38	0.00	0.00	0.01
	Threshold/criterion (*c*)						
	Race	1	17.25	< 0.001	0.03	0.01	0.06
	Writing task	2	0.89	0.41	0.00	0.00	0.01
	*df error*	518					
Model 2	Discriminability (*d'*)						
	Race	1	57.08	< 0.001	0.10	0.06	0.14
	Writing task	1	1.93	0.17	0.00	0.00	0.02
	Threshold/criterion (*c*)						
	Race	1	16.12	< 0.001	0.03	0.01	0.06
	Writing task	1	1.51	0.22	0.00	0.00	0.02
	*df error*	519					

Analyses revealed that individuals were significantly better at discriminating between armed and unarmed individuals among Black targets (*M* = 2.22, *SD* = 0.78) than among White targets, *M* = 1.99, *SD* = 0.71, *F*_(1, 518)_ = 70.17, *p* < 0.001, η*p*^2^ = 0.12, 95% CI [0.08, 0.16]. However, discrimination did not differ by writing task, *F*_(2, 518)_ = 0.962, η*p*^2^ = 0.004, 95% CI [0.00, 0.01]. In addition, participants showed significant bias in their shooting decisions: Participants had a significantly lower shooting criterion when presented with Black targets (*M* = −0.04, *SD* = 0.29) compared to White targets, *M* = 0.03, *SD* = 0.28, *F*_(1, 518)_ = 17.25, *p* < 0.001, η*p*^2^ = 0.03, 95% CI [0.01, 0.06]. However, bias was not predicted by writing task, *F*_(2, 518)_ = 0.892, η*p*^2^ = 0.003, 95% CI [0.00, 0.01].

Next, similar analyses were conducted using 2 (Writing task: Writing task vs. Control) × 2 (Race: Black vs. White) mixed models, with repeated measures on *race* to test whether *d'* or *c* varied for Black or White targets when collapsing across Writing Tasks. All patterns in our findings paralleled those, above (see Table 3).

### Shooting decision reaction time

To test whether the writing tasks impacted participants' shooting decision reaction time^2^ differentially for targets of different races who were armed or unarmed, we conducted a 3 (Writing Task: Purpose in Life vs. Self-Affirmation vs. Control) × 2 (Race: Black vs. White) × 2 (Weapon: Armed vs. Unarmed) mixed model analysis of variance, with repeated measures on the factors of race and weapon (see Tables 2, 4). No evidence was found of a three-way interaction, *F*_(2, 44, 712)_ = 0.19, *p* = 0.83.

**Table 4:** Effects of writing task, target race, and weapon presence of shooting decision reaction times.

				**Effect size**		
	***df***	***F***	***p***	*η^2^*	**90% CI**	
Writing task	2	2.81	0.06	0.00	0.00	0.00
Weapon	1	2,645.83	< 0.001	0.06	0.05	0.06
Race	1	2.90	0.09	0.00	0.00	0.00
Weapon × Race	1	28.38	< 0.001	0.00	0.00	0.00
Writing task × Weapon	2	1.19	0.30	0.00	0.00	0.00
Writing task × Race	2	0.19	0.83	0.00	0.00	0.00
Writing task × Weapon × Race	2	0.19	0.83	0.00	0.00	0.00
Error *df*	44,712					

Analyses revealed a significant main effect of weapon. Participants were significantly faster to shoot armed (*M* = 491.85, *SE* = 0.45) compared to unarmed (*M* = 525.49, *SE* = 0.48) targets, *t*_(44, 712)_ = −51.44, *p* < 0.001, *d* = 0.49, 95% CI [0.47, 0.51]. In addition, there was evidence of a significant interaction between race and weapon: Participants were significantly faster in their shooting decisions for armed Black (*M* = 490.66, *SE* = 0.63) compared to armed White targets, *M* = 493.03, *SE* = 0.64, *t*_(44, 712)_ = −2.64, *p* = 0.01, *d* = 0.03, 95% CI [0.01, 0.06]. Further, participants were significantly slower to decide that Black targets were unarmed (*M* = 527.79, *SE* = 0.68) compared to their decisions that White targets were unarmed [*M* = 523.19, *SE* = 0.66, *t*_(44, 712)_ = −4.84, *p* < 0.001, *d* = 0.07, 95% CI (0.04, 0.09)] (see Figure 1).

### Exploratory analyses

As discussed above, we found evidence to suggest that the purpose in life and self-affirmation writing tasks reduced the probability of shooting unarmed targets compared to individuals who wrote about a control topic. In an effort to determine by what mechanism purpose in life and self-affirmation offer protection against the improper shooting of unarmed targets, several exploratory analyses were conducted. First, a one-way analysis of variance (ANOVA) was used to examine whether the writing tasks influenced negative affect. However, analyses revealed that writing tasks were not predictive of either positive [*F*_(2, 502)_ = 0.77, *p* = 0.47, η^2^ = 0.00, 90% CI (0.00, 0.00)] or negative affect, *F*_(2, 509)_ = 0.61, *p* = 0.54, η^2^ = 0.00, 90% CI (0.00, 0.00).

Next, analyses were conducted to test whether the writing tasks differentially affected participants' internal and external motivations to not be prejudiced their colorblind racial attitudes, or their reliance on the moral foundations. First, difference scores were calculated by subtracting participants' pre-writing task scores on these measures from their post-writing task scores. Next, a one-way ANOVA was used to explore these relationships. Analyses revealed that writing task was not significantly predictive of changes in individuals' internal or external motivations not to be prejudiced or their colorblind racial attitudes, *ps* > 0.08 (see Table 5).

**Table 5:** Effect of writing task on individual difference measures.

				**Effect size**		
	***df***	***F***	***p***	*η^2^*	**90% CI**	
Positive affect	2,502	0.77	0.47	0.00	0.00	0.01
Negative affect	2,509	0.61	0.54	0.00	0.00	0.01
IMS	2,505	1.48	0.23	0.01	0.00	0.02
EMS	2,513	0.60	0.55	0.00	0.00	0.01
CoBRAS	2,454	0.55	0.58	0.00	0.00	0.01
Ryff	2,500	0.03	0.97	0.00	0.00	0.00
Care/harm	2,517	2.54	0.08	0.01	0.00	0.03
Fairness/cheating	2,517	4.18	0.02	0.02	0.00	0.04
Loyalty/betrayal	2,517	0.52	0.59	0.00	0.00	0.01
Authority/subversion	2,517	1.40	0.25	0.01	0.00	0.02
Sanctity/degradation	2,517	1.36	0.26	0.01	0.00	0.02

However, changes in individuals' reliance on the fairness moral foundation was predicted by Writing task, *F*_(2, 517)_ = 4.18, *p* = 0.02, η^2^ = 0.00, 90% CI [0.00, 0.00]. *Post-hoc* pairwise comparisons suggest that individuals who wrote about their purpose in life experienced a significant increase in their reliance on the fairness moral foundation (*M* = 0.05, *SE* = 0.03) compared to those who affirmed the self (*M* = −0.04, *SE* = 0.03, *p* = 0.04, *d* = 0.26, 95% CI [0.05, 0.49]) or wrote about a control topic, *M* = −0.05, *SE* = 0.03, *p* = 0.03, *d* = 0.28, 95% CI [0.06, 0.49].

**Discrimination and shooting decision criterion**. Next, exploratory analyses were conducted to test whether individuals' post-writing task scores on several individual difference measures (affect, reliance on the moral foundations, internal and external motivations to not be prejudiced, and colorblind racial attitudes) predicted differences in the ability to discriminate among armed and unarmed Black and White targets or individuals' shooting decision threshold. A linear regression testing the relationship between these individual difference measures and individuals' *d'* and *c* values were conducted separately for Black and White targets.

The overall model predicting discrimination among armed and unarmed White targets was significant. Positive affect was positively related to discrimination among White targets (*b* = 0.01, *SE* = 0.00, *t* = 5.11, *p* < 0.001), whereas negative affect was negatively related, *b* = −0.01, *SE* = 0.00, *t* = −2.91, *p* = 0.004, *F*_(11, 426)_ = 4.24, *p* < 0.001, *R*^2^ = 0.10, 95% CI [0.03, 0.13]. Similarly, positive affect was positively related to discrimination among Black targets (*b* = 0.01, *SE* = 0.00, *t* = 3.44, *p* = 0.001), whereas negative affect was negatively related, *b* = −0.01, *SE* = 0.00, *t* = −2.28, *p* = 0.02, *F*_(11, 426)_ = 2.22, *p* = 0.01, *R*^2^ = 0.05, 95% CI [0.00, 0.08]. However, linear regression analyses revealed that these individual difference measures were unrelated to participants' shooting decision criterion for White [*F*_(11, 426)_ = 0.70, *p* = 0.74, *R*^2^ = 0.02, 95% CI (0.00, 0.02)] and Black targets, *F*_(11, 426)_ = 0.90, *p* = 0.54, *R*^2^ = 0.02, 95% CI [0.00, 0.03].

## Discussion

The current study examined the impact of purpose in life and self-affirmation writing tasks on basic psychological processes that might relate to the decision to shoot and shooting decision accuracy utilizing a first-person shooter video game for Black compared to White targets, and second, investigated whether the writing tasks might have a differential impact on such processes. Partial support was found for our first hypothesis: Findings provide initial evidence that purpose in life and self-affirmation improved shooting decision accuracy, though this effect did not vary by target race. Specifically, writing about one's purpose in life or self-affirming values was related to a reduction in the improper shooting of unarmed individuals in a first-person shooting video game, regardless of race. It is important to note that, although purpose in life and self-affirmation reduced the probability of shooting unarmed targets, this reduction was not accompanied by a tradeoff in shooting decision reaction time: Shooting decision reaction time did not differ by writing task, and no significant interactions were found between writing task and weapon nor between writing task and race. Thus, it appears purpose in life and self-affirmation can offer benefits in shooting decision accuracy in this context without sacrificing quick responses in the face of threat. These findings have important implications for policing reforms because it shows how basic psychological processes can contribute to the mitigation of police shootings of unarmed individuals, thereby providing insight into the psychological mechanisms that could be harnessed in the search for solutions to the problem. These results suggest that police might benefit from writing about their purpose in life or self-affirming values.

Writing about one's purpose in life or self-affirming values reduced the probability of shooting unarmed suspects in a first-person shooter video game. In the current context, we found no evidence of a *differential* effect of these writing tasks on shooting decisions. In the field, officers who confront armed or unarmed suspects may experience both physical and existential threat: Officers may fear for their physical safety and may be motivated to avoid appearing prejudiced. In the current study, purpose in life and self-affirmation may both have contributed to a reduction in perceptions of existential threat and defensiveness. Both writing tasks target existential threat, but in doing so, both may in turn reduce perceptions of physical threat and defensiveness, thereby improving shooting decisions for unarmed suspects. Further, both have been shown to reduce racial biases and improve comfort with diversity, which could explain why there was improvement in accurate shooting decisions for both Black and White unarmed targets, given past findings demonstrating that participant shooting decision accuracy is lower for unarmed Black compared to White targets (e.g., Correll et al., 2002). However, in the present study, neither writing intervention predicted participant internal or external motivations to respond without prejudice, suggesting that racial bias reduction may not have played a central role in the improvement in shooting decision accuracy in this case. This may suggest other mechanisms are at play, including metacognitive processes (Owens-Boone, 2023).

By what mechanism both purpose in life and self-affirmation writing tasks reduced the shooting of unarmed targets is unclear. Neither purpose in life nor self-affirmation predicted positive or negative affect, though on the whole, positive affect was associated with shooting decision discrimination, and negative affect negatively related, respectively. Prior research demonstrates some success in the use of mood induction as a tool for bias reduction (for a review, see FitzGerald et al., 2019). Given that the writing tasks tested in the current study were unrelated to affect, future research should further investigate the possibility of a connection between such tasks, affect, and bias reduction.

Further, though participants who engaged with the purpose in life writing task showed higher post-intervention scores on the fairness moral foundation compared to those who affirmed the self or wrote about a control topic, fairness did not predict differences in participants' ability to discriminate among targets, nor did it predict shooting criterion. These findings stand in contrast to research demonstrating success in reducing bias via interventions that encourage tolerance and respect (Blincoe and Harris, 2009) and those priming participants with concepts from Buddhism (Clobert et al., 2015). As the writing tasks in the current research did not reduce prejudice, future research must investigate by what mechanism cultivating purpose in life and affirming the self reduced the shooting of unarmed targets broadly.

Discrepancies between past and present findings may be explained by the broad discretion of purpose in life and self-affirmation writing interventions. Unlike other interventions that have reduced racial bias (for a review, see FitzGerald et al., 2019; see also Lai et al., 2014), the writing tasks of the current research offer participants the opportunity to write about *any* life purpose that they may have, or *any* importantly held value. In contrast, other research asks participants to more specifically target particular themes (e.g., Clobert et al., 2015), or more directly target stereotypical beliefs (e.g., Woodcock and Monteith, 2013). Thus, the purposes and/or values addressed by participants in the current research likely vary widely, making it difficult to pinpoint by what mechanism these interventions reduced the shooting of unarmed targets.

Replicating prior research, participants were more likely to shoot armed compared to unarmed individuals (e.g., Correll et al., 2002, 2006; Greenwald et al., 2003). Participants were also more likely to shoot Black compared to White men, which is consistent with related research suggesting that police officers are more likely to use force against male minorities compared to other groups (e.g., Bolger, 2015). However, unlike prior research, no racial differences were found for shooting decisions regarding unarmed targets using similar methods (e.g., Correll et al., 2002). Findings from the current study instead found racial differences in the shooting of armed targets, such that participants were more likely to shoot armed Black compared to armed White targets. The current study was highly powered. In addition, measuring motivations to respond without prejudice and participants' color-blind racial attitudes before and after the completion of the shooter task may have primed participants to be particularly attentive when making shooting decisions regarding unarmed suspects. However, the current results suggest participants made shooting decision errors (refraining from shooting White targets that were armed), evidencing bias.

### Limitations and future directions

The current study is not without limitations. While it is important to examine the impact of these writing tasks in a tightly controlled laboratory environment, these findings may not generalize to officers in the field tasked with making life or death decisions. Further, the first-person shooter video game used in the current study is of course dissimilar to officers' environments within the field. However, the current study makes an important contribution to a growing body of work aimed at improving shooting decisions in police use of force.

Further, the mechanism by which writing about purpose in life or self-affirming values impacted decisions to shoot remains unclear. While writing about one's purpose in life increased participants' reliance on Fairness relative to the control and self-affirmation writing tasks, Fairness was unrelated to participants' ability to discriminate among armed and unarmed targets for either Black or White targets and was unrelated to participants' shooting criterion. Further, positive affect was positively related and negative affect negatively related to the ability to discriminate between armed and unarmed targets for White and Black targets. However, neither positive nor negative affect was related to participants' shooting criterion for Black or White targets, and further, neither purpose in life nor self-affirming beliefs were related to changes in positive or negative affect. Lastly, neither writing task was related to changes in internal and external motivations to not be prejudiced, nor color-blind racial attitudes, relative to the control and one another. Thus, the mechanism by which writing about one's purpose in life or self-affirming values reduced the shooting of unarmed targets relative to writing about a control topic remains unknown. Future research must continue to examine by what mechanism such writing interventions improved shooting decision accuracy, and whether these psychological processes (e.g., positive affect) might be supportive in the field. In addition, scholars might consider the content of individuals' writing when intervening with purpose in life or self-affirmation writing tasks.

But by what mechanism did purpose in life and self-affirmation protect against the shooting of unarmed targets? One possibility is that writing about one's purpose in life or affirming the self increased cognitive control and/or reduces impulsivity. Related research suggests that “shooter bias” is less pronounced in individuals with high cognitive control (Payne, 2005), and Correll et al. (2006) argue that the key to reducing improper shootings is response inhibition, though the current study revealed an improvement in shooting decision accuracy for unarmed targets, regardless of race. Purposeful individuals are less impulsive compared to those lacking purpose (Burrow and Spreng, 2016), and other work suggests self-affirmation is related to self-regulation and may help to combat depletion (Loseman and van den Bos, 2012; Schmeichel and Vohs, 2009). Research generally suggests that purpose in life speeds up one's recovery from stressful situations (e.g., Fogelman and Canli, 2015; Schaefer et al., 2013). Further, purposeful individuals experience less anxiety, somatic symptoms, and sympathetic nervous system activation in response to anxiety and fear-provoking stimuli compared to less purposeful individuals, while also experiencing greater parasympathetic nervous system activation in response to such stressful stimuli compared to less purposeful individuals (Ishida and Okada, 2006). Future research should further investigate by what mechanism these two constructs improve shooting decisions for unarmed targets in this context, and whether such writing tasks might be helpful to officers in the field, including the relationship between these writing tasks and other measures of stress (e.g., physiological responses) and perceptions of threat.

Importantly, the current research can only speculate about the role of threat and threat reduction as they may relate to the tested writing interventions, limiting our ability to make direct inferences about threat and threat reduction in this context as this construct was not directly measured. Given the behavioral tasks and measures used in the current research, and the desire to pursue novel research questions using a validated behavioral task, we were unable to assess perceptions of threat. Ideally, threat would be assessed for each trial, given that perceived threat likely varies dependent upon weapon status (whether the target is armed or unarmed), and for some, dependent upon target race. Assessing perceptions of threat for each trial would likely have been disruptive for participants, so in the current study, tradeoffs were made between ecological validity, demand characteristics, and the desire to use a validated behavioral task. However, future research should consider various momentary measures of perceived threat, including physiologic responses (e.g., galvanic skin response, heart rate variability; see Meehan et al., 2002).

There are noticeable gaps in our current knowledge surrounding self-affirmation and purpose in life, and how they might apply to decision making and behavior. We do not fully understand the mechanisms by which these constructs benefit us, or how they differ in terms of the processes by which they help us to reap such benefits. Research demonstrates that such writing tasks are not always sufficient to impact human behavior (e.g., Vohs et al., 2013). Thus, more research is needed to determine the link between one's intentions and their ability or willingness to act on such intentions.

## Conclusion

The current study investigated the influence of purpose in life and self-affirmation on shooting decisions in a first-person shooter video game. This work provides initial insight into the psychological processes that might be harnessed in the context of policing that may increase shooting decision accuracy for unarmed individuals. Future research should investigate the potential impact of these constructs within officers in the field, and continue to investigate whether writing interventions might reduce racial biases in this context. Further, scholars should also examine whether such writing tasks have other positive downstream effects (e.g., overall decreases in stress, improvement in self-esteem, etc.) that might have a recursive effect on shooting decisions.

## Data Availability

The raw data supporting the conclusions of this article will be made available by the authors, without undue reservation.

## Footnotes

^1^Nineteen participants (~3.7%) completed the experiment across two experimental sessions. In the first session, participants completed the individual difference measures. For these individuals, participation in the second session of the experiment occurred on average 11.15 days after the first session. During the second session, individuals began by completing the writing task and then participated in the first-person shooter video game. After, they completed the individual difference measures for a second time along with the demographic questions. An exploratory model was conducted, with session number entered as main and interactive terms and shooting decision as the outcome. There was no evidence of a main effect, nor an interaction with writing task, *ps* ≥ 0.33.

^2^It is important to note that the reaction time data could be approached using several methods, with important considerations for each. For one, if the writing tasks led to longer reaction times compared to the control condition, reflecting, albeit indirectly, an increase in cognitive control, then one option would be to use log-transformed reaction times as a covariate in our model predicting shooting decisions. The difficulty in using this method in the current paradigm is that participants' trials were omitted when they extended past 630 ms. Though we cannot know for sure whether participants would have made correct or incorrect decisions to shoot armed targets and to choose not to shoot unarmed targets after this time, when a target was unarmed, this delay may ultimately have led to a correct decision not to shoot, and thus, reaction times are confounded with these missing data from trials in which the participant timed out. As such, we chose to analyze shooting decisions and reaction time data separately.
